# Changes in kidney function after adrenalectomy in patients with primary aldosteronism

**DOI:** 10.20407/fmj.2024-011

**Published:** 2024-10-31

**Authors:** Yumi Tomiie, Yatsuka Hibi, Rie Nobe, Keito Yokoi, Yusuke Koshima, Kimio Ogawa, Tsuneo Imai, Zenichi Morise

**Affiliations:** 1 Department of Endocrine Surgery, Fujita Health University, School of Medicine, Toyoake, Aichi, Japan; 2 Department of Nurse Practitioner, Fujita Health University Hospital, Toyoake, Aichi, Japan; 3 Department of Surgery, Fujita Health University, School of Medicine, Okazaki, Aichi, Japan

**Keywords:** Primary aldosteronism, Adrenalectomy, Estimated glomerular filtration rate, Kidney dysfunction, Hypokalemia

## Abstract

**Objectives:**

A decrease in the estimated glomerular filtration rate (eGFR) is occasionally observed in patients with primary aldosteronism (PA) after adrenalectomy. Patients may misunderstand that the surgical stress of adrenalectomy can result in kidney dysfunction. However, this finding is considered due to postoperative manifestations of kidney dysfunction that are masked preoperatively by excess aldosterone. To evaluate kidney dysfunction unmasked by adrenalectomy, we investigated changes in the eGFR after adrenalectomy according to the clinically assessable indication of “a certain drop in eGFR” as defined by the 2012 Kidney Disease Improving Global Outcomes clinical practice guideline.

**Methods:**

This study included 54 patients with PA who underwent unilateral adrenalectomy between 2005 and 2022 at our institution. We classified patients by GFR categories defined by the guideline according to their pre- and postoperative eGFR. We analyzed the predictors associated with a certain drop in eGFR (i.e., a decrease in GFR category accompanied by a ≥25% decrease in the eGFR from baseline).

**Results:**

A certain drop in eGFR was present in 35.2% of patients after adrenalectomy. Multivariate regression analysis showed that a longer duration of hypertension, lower preoperative serum potassium concentrations, and lower serum potassium concentrations before potassium supplementation were significant independent predictors (*p*<0.05). The cut-off value of the preoperative serum potassium concentrations was 3.7 mmol/L according to receiver operating characteristic curve analysis.

**Conclusions:**

Our findings will be useful for surgeons in informing patients with PA regarding the possibility of downgrading GFR categories after adrenalectomy.

## Introduction

Primary aldosteronism (PA) is a disease characterized by autonomous hypersecretion of aldosterone and induces secondary hypertension as a result of excessive sodium retention and potassium excretion. The two primary causes of PA are aldosterone-producing adenoma and bilateral adrenal hyperplasia, also known as idiopathic hyperaldosteronism. Aldosterone-producing adenoma is the most prevalent cause of PA and is usually located unilaterally in the adrenal gland. Adrenalectomy is the preferred treatment for patients with unilateral aldosterone-producing adenoma, improving or even normalizing secondary hypertension in the majority of patients.

Hypersecretion of aldosterone promotes sodium reabsorption in the distal tubules of the kidney and an increase in extracellular volume, leading to secondary hypertension.

This sequence of events increases perfusion of the kidney and results in glomerular hyperfiltration, which can mask a preoperative decrease in the estimated glomerular filtration rate (eGFR). Furthermore, long-term exposure to excess aldosterone damages kidney cells directly and causes a decrease in the eGFR.^1,2^ Previous studies have shown that patients with PA often have a considerable decrease in eGFR after unilateral adrenalectomy.^3,4^ This reduction in the eGFR just after adrenalectomy may give the impression that the surgery itself induces kidney dysfunction, but it is thought to be caused by hyperfiltration resulting from excess aldosterone and to result in unmasking of the original PA-induced kidney dysfunction.^1^ In patients who expect adrenalectomy to improve their condition, the appearance of postoperative kidney dysfunction may be misinterpreted as a surgical complication. Surgeons need to predict these changes in perioperative kidney function and explain them to patients before surgery. Additionally, if more severe kidney impairment is concealed in patients with PA, the prognosis of these patients would be even poorer than predicted.

The 2012 Kidney Disease Improving Global Outcomes (KDIGO) Clinical Practice Guideline for the Evaluation and Management of Chronic Kidney Disease classifies chronic kidney disease (CKD) by the GFR. The GFR category is assigned based on the eGFR as follows: G1, normal or high (eGFR ≥90 mL/min/1.73 m^2^); G2, mildly decreased (60–89 mL/min/1.73 m^2^); G3a, mildly to moderately decreased (45–59 mL/min/1.73 m^2^); G3b, moderately to severely decreased (30–44 mL/min/1.73 m^2^); G4, severely decreased (15–29 mL/min/1.73 m^2^), and G5, kidney failure (<15 mL/min/1.73 m^2^).^5^ Furthermore, the guideline defines “… a certain drop in eGFR; a drop in GFR category accompanied by a 25% or greater drop in eGFR from baseline” as one of the criteria for progression of CKD.^5^ The guideline also states that evaluation of the GFR category and progression of CKD can predict all-cause and cardiovascular mortality.^5,6^

There is no established method for evaluating changes in kidney function before and after adrenalectomy in patients with PA. In this study, we considered the changes in kidney function that occur before and after adrenalectomy as unmasked manifestations of the chronic effects of excess aldosterone and evaluated them according to CKD guidelines. In addition, when surgeons perform adrenalectomy for PA, they are expected to predict perioperative changes in kidney function under conditions in which the patient with PA has already been started on potassium supplementation, various antihypertensive drugs and mineralocorticoid receptor (MR) antagonists. We aimed to identify factors that can predict a certain drop in eGFR after adrenalectomy from a surgeon’s perspective based on perioperative real-world data from patients with PA.

## Methods

### Study population

We retrospectively reviewed the medical records of 85 patients with PA who underwent unilateral adrenalectomy at Fujita Health University Hospital between January 2005 and December 2022. Thirty-one patients were excluded because of insufficient data or too short a follow-up period (<1 year). Finally, 54 patients were enrolled (see Supplementary Figure).

### Diagnosis of PA

PA was basically screened by using the aldosterone-to-renin ratio and diagnosed by a positive result in one or more of the following: the captopril challenge test, the furosemide upright test, and the saline infusion test. Although eight patients did not undergo these confirmatory tests owing to kidney dysfunction or severe hypokalemia, they were suspected of having PA on the basis of an aldosterone-to-renin ratio >200 and underwent a detailed examination. Abdominal computed tomography (CT) or magnetic resonance imaging was performed in all patients to confirm tumor localization. The CT/magnetic resonance imaging findings showed that 46 patients had a unilateral mass lesion, 5 had unclear lesions, and 3 had bilateral mass lesions. In 47 patients, adrenal venous sampling (AVS) was also performed for functional localization in accordance with the Japan Endocrine Society guidelines.^7^ Patients with unclear or bilateral lesions were diagnosed as having a unilateral lesion by AVS. Three patients were diagnosed to have a unilateral mass lesion on the basis of CT findings but AVS showed a functional lesion of the contralateral adrenal gland.

In seven patients who did not undergo AVS, adrenocortical scintigraphy with dexamethasone suppression was performed using NCL-6-^131^I (marker of adrenocortical cholesterol uptake). Dexamethasone 4 mg/day orally was started 7 days before injection of NCL-6-^131^I and continued until the last day of scanning to improve the diagnostic usefulness of this test by suppression of glucocorticoid synthesis. The unilateral lesions were diagnosed on the basis of concordance of the mass lesions identified by CT and the scintigraphic uptake. Finally, all 54 patients were diagnosed as having a unilateral functional lesion, which was an indication for surgery. All patients underwent unilateral adrenalectomy (laparoscopic, *n*=52; open retroperitoneal approach, *n*=2) depending on the result of localization studies (see Supplementary Figure).

### Measurement of clinical data

Collected clinical data included age, sex, body mass index (calculated as kg/m^2^), duration of hypertension, systolic and diastolic blood pressure, and antihypertensive drugs (except for MR antagonists), MR antagonists (spironolactone or eplerenone), and potassium supplementation used before surgery. In addition, smoking, diabetes mellitus, and hyperlipidemia were investigated as factors affecting kidney function. Laboratory data, such as concentrations of plasma aldosterone, serum sodium, serum potassium, serum creatinine, and serum total cholesterol, the eGFR, and plasma renin activity, were collected shortly before adrenalectomy to compare kidney function before and after surgery. Serum potassium concentrations were evaluated not only immediately before adrenalectomy but also preoperative serum potassium concentrations before potassium supplementation. The reason for choosing these time points is that many patients had already received direct preoperative therapeutic intervention for hypokalemia. The duration of hypertension was defined as the period between when the patient was diagnosed with hypertension or when antihypertensive treatment was initiated (ascertained by an interview with the patient) and the time of adrenalectomy. The eGFR values were obtained using the following equation: eGFR (mL/min/1.73 m^2^)=194×(serum creatinine)^–1.094^×(age)^–0.287^×0.739 (if female).^8^ All measurements of plasma aldosterone concentration were performed by radioimmunoassay.

We investigated eGFR levels at 1 day, 1 week, 2 weeks, 1 month, 3 months, 6 months, and 1 year after adrenalectomy, and evaluated changes during that time. We also evaluated “a certain drop in eGFR” by eGFR levels and GFR category at 1 year after adrenalectomy and compared the group of patients with a certain drop in eGFR and those without a certain drop in eGFR. A certain drop in eGFR was defined according to the KDIGO guideline definition as a decline in the GFR category with a ≥25% drop in eGFR from the preoperative value.^5^

### Statistical analysis

Continuous variables are expressed as the mean±standard deviation or the median (range) and categorical variables as the number (percentage) as appropriate. The patients were divided into a group with a certain drop in eGFR and a group without a certain drop in eGFR. Continuous variables were compared between the two groups using Student’s *t*-test and categorical variables were compared using the chi-squared test. Non-parametric variables were compared using the Mann–Whitney *U* test. Multiple comparisons with Tukey’s test were used to compare changes in the eGFR before and after adrenalectomy.

Predictors associated with a certain drop in eGFR were investigated in univariate and multivariate logistic regression analyses. The statistical analyses were performed using IBM SPSS version 28 (IBM Corp., Armonk, NY, USA) except for the receiver operating characteristic (ROC) curve analysis, which was performed using EZR analysis software version 1.55 (Saitama Medical Center, Jichi Medical University, Saitama, Japan)^9^ to set and evaluate the cut-off value. A *p* value <0.05 was considered statistically significant.

### Ethics statement

The study was approved by the institutional ethics review committee at Fujita Health University School of Medicine (HM20-443) and conducted in accordance with the guidelines outlined in the 1964 Declaration of Helsinki and subsequent revisions. This was a retrospective, noninterventional study with use of existing data. Therefore, informed consent was obtained via the opt-out route in accordance with the ethical guidelines defined by the Japanese Ministry of Health, Labour and Welfare.

## Results

### Clinical outcomes of adrenalectomy

All 54 patients showed normalization of their plasma aldosterone concentrations (<240 pg/mL, measured by radioimmunoassay) and/or the aldosterone-to-renin ratio (<200). Consequently, hypertension improved in 52 of 54 patients. All 33 patients who required oral potassium supplementation preoperatively were able to discontinue it after adrenalectomy. A pathological examination resulted in 51 of 54 patients being clearly diagnosed with adrenocortical adenoma.

### Patients’ characteristics

The clinical characteristics of the 54 eligible patients are presented in Table 1. Compared with the group without a certain drop in eGFR (*n*=35; 64.8%), the group with a certain drop in eGFR (*n*=19; 35.2%) had a significantly longer mean duration of hypertension (*p*=0.026), lower mean preoperative serum potassium concentration (*p*=0.009), and lower mean serum potassium concentration before potassium supplementation (*p*=0.001). Eighteen (33.3%) patients were taking an angiotensin II receptor blocker/angiotensin-converting enzyme inhibitor preoperatively and 15 (27.8%) patients were taking an MR antagonist preoperatively. However, there was no significant difference in the frequency of taking these drugs between the two groups. In addition, 11 (20.4%) patients had diabetes and none of them were taking a sodium-glucose transport protein 2 inhibitor. There was no significant difference in the presence or absence of diabetes between the two groups.

### Changes in kidney function after adrenalectomy

**Figure 1:**
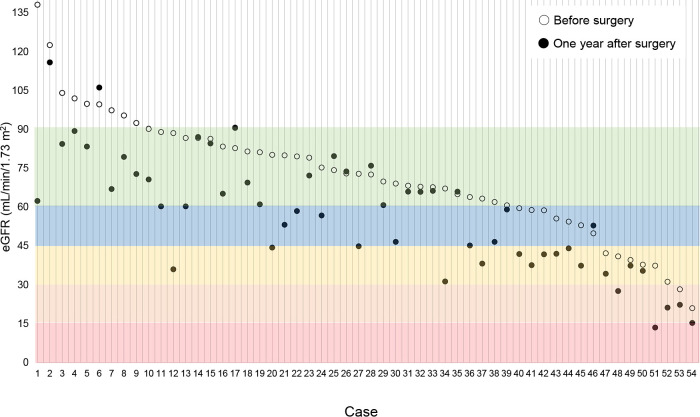
Changes in the eGFR in each patient Case numbers are allotted in order according to the preoperative eGFR. White, green, blue, yellow, orange, and red zones represent the G1, G2, G3a, G3b, G4, and G5 categories, respectively. The postoperative eGFR was recorded at 1 year after adrenalectomy. eGFR, estimated glomerular filtration rate

**Figure 2:**
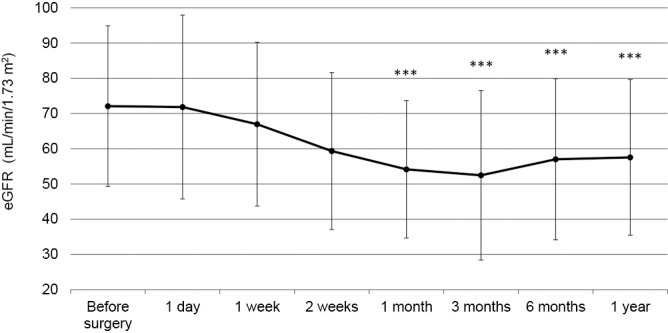
Changes in the eGFR after adrenalectomy The eGFR at 1 month after adrenalectomy was significantly lower than that preoperatively. There was no significant difference in the eGFR between 1 month postoperatively and subsequent months. The data are shown as the mean and standard deviation. ***p<0.05 vs preoperative eGFR. eGFR, estimated glomerular filtration rate

**Figure 3:**
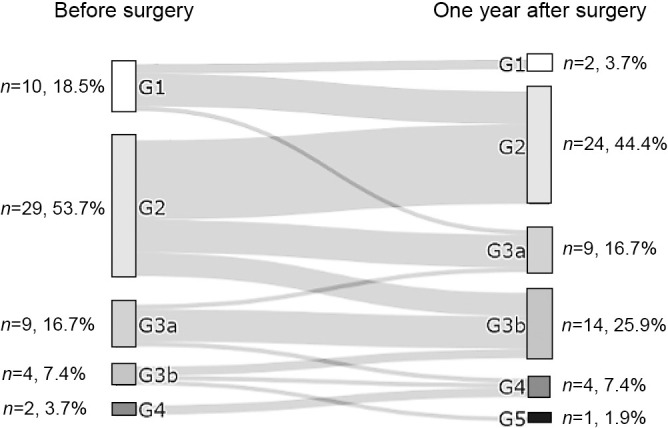
Distribution of GFR categories before and 1 year after adrenalectomy The proportion of patients with an eGFR <60 mL/min/1.73 m^2^ (G3 or higher category, classified as chronic kidney disease) was approximately one third (27.8%) preoperatively and more than half at 1 year postoperatively.

**Figure 4:**
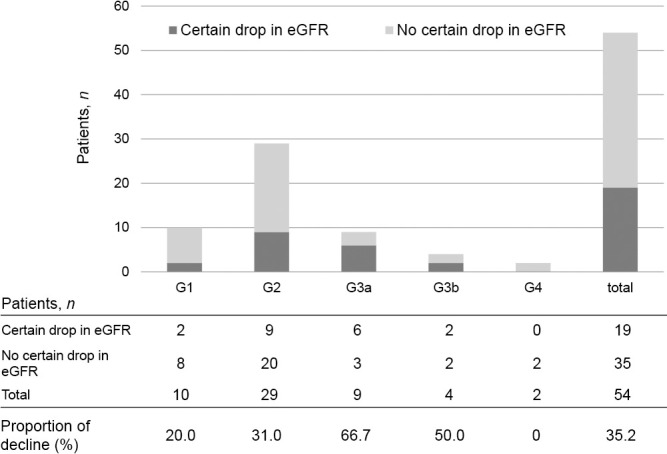
Proportion of certain drop in eGFR 1 year after adrenalectomy according to the preoperative GFR category There was no significant difference in the proportion of patients with a certain drop in eGFR for each GFR category (*p*=0.157). eGFR, estimated glomerular filtration rate

Changes in the eGFR are shown for each patient in Figure 1. The mean eGFR at 1 year postoperatively was significantly lower than the mean preoperative eGFR (57.5±22.0 vs 72.1±22.7 mL/min/1.73 m^2^, *p*=0.001). Furthermore, the eGFR at 1 month after adrenalectomy was significantly lower than that preoperatively (54.1±19.5 vs 72.1±22.7 mL/min/1.73 m^2^, *p*<0.001). The eGFR was lowest at 3 months after adrenalectomy. However, there was no significant difference in the eGFR between 1 month postoperatively and subsequent months (Figure 2). Figure 3 shows the transition of GFR categories from preoperatively to 1 year postoperatively. A G3 or higher category, which is CKD, accounted for approximately 30% of the total, but this increased to more than half of the total postoperatively. Figure 4 shows the proportion of a certain drop in eGFR by 1 year after adrenalectomy for each preoperative GFR category. The certain drop in eGFR was 35.2% in total, with the highest proportion observed in the G3a category. There was no significant difference in the proportion of patients with a certain drop in eGFR in each GFR category (*p*=0.157).

### Factors associated with a certain drop in eGFR

We performed univariate and multivariate logistic regression analyses including factors that were significantly different between the groups with a certain drop in eGFR and without a certain drop in eGFR (Table 2). In the univariate analysis, the duration of hypertension (odds ratio [OR] 1.082, 95% confidence interval [CI] 1.006–1.163, *p*=0.035), preoperative serum potassium concentrations (OR 0.203, 95% CI 0.057–0.731, *p*=0.015) and serum potassium concentrations before potassium supplementation (OR 0.124, 95% CI 0.030–0.516, *p*=0.004) were significant factors associated with a certain drop in eGFR. In the multivariate analysis, the duration of hypertension (OR 1.173, 95% CI 1.043–1.319, *p*=0.008), preoperative serum potassium concentrations (OR 0.145, 95% CI 0.026–0.818, *p*=0.029) and serum potassium concentrations before potassium supplementation (OR 0.111, 95% CI 0.017–0.731, *p*=0.022) were also significant independent factors associated with a certain drop in eGFR. Therefore, a longer duration of hypertension, lower preoperative serum potassium concentrations and serum potassium concentrations before potassium supplementation were associated with an increased risk of a certain decline in the eGFR.

### Cut-off values of serum potassium concentrations associated with a certain drop in eGFR

**Figure 5:**
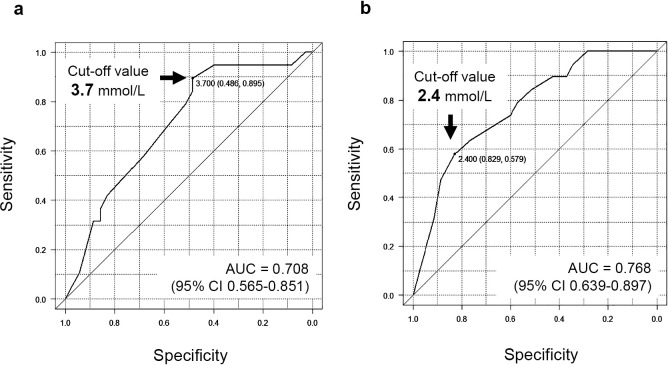
Cut-off values for serum potassium concentrations associated with a certain drop in eGFR a. The cut-off value for preoperative serum potassium concentrations related to a certain drop in eGFR was 3.7 mmol/L (sensitivity, 89.5%; specificity, 48.6%). The AUC was 0.708 (95% CI 0.565–0.851). b. The cut-off value for serum potassium concentrations before potassium supplementation related to a certain drop in eGFR was 2.4 mmol/L (sensitivity, 57.9%; specificity, 82.9%). The AUC was 0.768 (95% CI 0.639–0.897). eGFR, estimated glomerular filtration rate; AUC, area under the receiver operating characteristic curve; CI, confidence interval

We selected the optimal cut-off value using ROC curve analysis to identify serum potassium concentrations associated with a certain drop in eGFR (Figure 5). Preoperative serum potassium concentrations and serum potassium concentrations before potassium supplementation were analyzed. The cut-off value of preoperative serum potassium concentrations related to a certain drop in eGFR was 3.7 mmol/L (sensitivity, 89.5%; specificity, 48.6%). The area under the ROC curve was 0.708 (95% CI 0.565–0.851) (Figure 5a). The cut-off value of serum potassium concentrations before potassium supplementation related to a certain drop in eGFR was 2.4 mmol/L (sensitivity, 57.9%; specificity, 82.9%). The area under the ROC curve was 0.768 (95% CI 0.639–0.897) (Figure 5b).

## Discussion

In this study, we examined real-world data on changes in kidney function before and after adrenalectomy from the perspective of surgeons who perform adrenalectomy for PA. As previously reported,^1,3,10–12^ this study showed a decreased eGFR in patients with PA after adrenalectomy. Furthermore, we were able to categorize this decrease in the eGFR using the method recommended by the 2012 KDIGO clinical practice guideline and showed how each category of kidney function changed after adrenalectomy. Previous studies have investigated the extent of the decrease in the eGFR and the percentage of patients with CKD after adrenalectomy.^13–15^ To the best of our knowledge, no previous study has examined these GFR categories and how they change after adrenalectomy.

Several recent studies showed a decrease in the eGFR within the first month after adrenalectomy without any further change over time.^11,13,15–17^ Kim et al. compared postoperative changes in the eGFR at 3 days, 2 weeks, and 6 months after adrenalectomy in patients with and without PA.^1^ They found that patients with PA had a significantly lower postoperative eGFR than those without PA at all time points. Furthermore, there was no significant difference in the decrease in the eGFR between 2 weeks and 6 months after adrenalectomy in patients with PA.^1^ We found a significant decrease in the eGFR at 1 month after adrenalectomy compared with the preoperative value. Furthermore, the eGFR was decreased to 52.4 mL/min/1.73 m^2^ at 3 months and was increased to 57.0 mL/min/1.73 m^2^ at 6 months and to 57.5 mL/min/1.73 m^2^ at 1 year after adrenalectomy. However, no significant difference in the eGFR was observed between 1 month after adrenalectomy and 1 year postoperatively. Our data indicate that glomerular hyperfiltration caused by aldosterone excess may resolve within 1 month after adrenalectomy. The prevalence of CKD before adrenalectomy was 27.8% in this study, which is higher than that in other reports.^1,14^ Treatment with MR antagonists has been reported to decrease the eGFR similarly to adrenalectomy in patients with PA.^18^ Although the effect of including 15 (27.8%) patients who had received preoperative medical therapy with MR antagonists to compare pre- and post-adrenalectomy should be considered, our findings suggested that the presence or absence of treatment with MR antagonists was not a significant factor associated with a certain drop in eGFR.

We also found that 33.3% of patients with G1 or G2 progressed to G3 or G4, which was defined as CKD, 1 year after adrenalectomy. Several other studies investigated factors that predict development of CKD after adrenalectomy. Kim et al. investigated 89 patients with PA and identified old age, low serum potassium concentrations, a low preoperative eGFR, and high serum cholesterol concentrations as independent predictors of postoperative CKD.^1^ Other studies have also shown that low serum potassium concentrations are associated with a decreased eGFR after treatment.^15,18^ We found that preoperative serum potassium concentrations (including patients on potassium supplementation) and serum potassium concentrations before potassium supplementation were significant risk factors for a certain drop in eGFR. Reungjui et al. reported that chronic hypokalemia caused kidney injury via tubular hyperplasia, cell proliferation, macrophage infiltration, and interstitial fibrosis.^19^ This finding is consistent with our results. In our study, the ROC curve analysis suggested that attention should be paid to a decline in postoperative kidney function, especially if serum potassium concentrations before potassium supplementation are <2.4 mmol/L. Moreover, even if preoperative potassium correction is performed for hypokalemia, the same caution should be exercised when preoperative serum potassium concentrations are <3.7 mmol/L. Although several reports have shown that hypokalemia is a factor involved in decreased kidney function after adrenalectomy,^1,15,18^ this is the first report to specifically examine serum potassium concentrations. In addition, we found no significant difference in the presence or absence of preoperative potassium supplementation when we compared the groups with and without a certain drop in eGFR. This finding could be useful for surgeons because it suggests that serum potassium concentrations can be a predictive factor for changes in kidney function, even if patients have undergone potassium correction before adrenalectomy.

Several recent studies have shown that the duration of hypertension is a factor involved in a decrease in the postoperative GFR, which is consistent with our findings.^1,17,18^ Chronic hypertension caused by aldosterone excess and the direct effects of aldosterone result in structural injury to the kidney and cardiovascular tissues.^1,2,20,21^ Excess aldosterone has specific effects on the kidney, causing glomerular hypertension via its vasoconstrictive effect on the glomerular arterioles, which results in structural damage to the glomerulus.^22^ A longer duration of disease, such as exposure to excess aldosterone and hypertension, is a risk factor for kidney dysfunction. Chen et al. compared the prognosis of patients treated for essential hypertension with that of those who underwent adrenalectomy for PA and reported a lower risk of end-stage kidney disease after adrenalectomy.^23^ This report and our findings support the importance of early diagnosis of PA and resolution of the secondary hypertension of excess aldosterone as soon as possible to prevent progression of kidney dysfunction.

There is concern that surgery in patients with severe kidney dysfunction may trigger early postoperative introduction of dialysis. However, in our study, two patients in the G4 did not show a certain drop in eGFR although there was a certain drop in eGFR in each category. Utsumi et al. reported that kidney function decreases beyond a certain limit, fibrosis of the intrarenal vessels reduces perfusion in the glomerulus, and glomerular hyperfiltration is attenuated.^14^ Patients with severe kidney dysfunction above G4 are thought to have a severe degree of glomerular fibrosis due to excess aldosterone and no longer show a decline in the eGFR that corresponds to changes in aldosterone secretion. In our series, the two patients in G4 did not require maintenance dialysis until 2.5 years and 4.5 years after adrenalectomy. On the basis of these considerations, adrenalectomy may also be indicated for PA patients with severe kidney impairment who have not yet initiated maintenance dialysis.

The long-term effect of changes in kidney function after adrenalectomy on patients with PA in this study is an important clinical issue. Even if there is a decline in the eGFR in the short postoperative period, removing excess aldosterone may ultimately preserve a patient’s kidney function better than not operating. However, there have been few studies on the long-term postoperative prognosis of the kidney after adrenalectomy for PA. Chen et al. reported that the risk of long-term adverse kidney events increased with a decline in the eGFR of more than 30% at 6 months after adrenalectomy, although it did not affect mortality.^12^ In contrast, Kobayashi et al. reported no association between the degree of decline in the eGFR at 6 months after adrenalectomy for PA and the slope of a long-term decline in the eGFR.^24^ Therefore, the long-term kidney prognosis after adrenalectomy is currently controversial.

The limitations of this study include its retrospective design, the relatively small number of patients, the short follow-up period, and the method of evaluating postoperative kidney function. In this study, we investigated the 1-year follow-up after adrenalectomy but the long-term effects on kidney function and prognosis of survival were not determined. Further long-term follow-up is required because the long-term kidney prognosis is unclear. Another possible limitation is that an early postoperative decline in the eGFR may indicate acute kidney injury by surgical treatment, and the assessment by CKD progression alone might be insufficient. The 2012 KDIGO clinical practice guideline also classifies CKD on the basis of albuminuria. However, few patients in our series were evaluated for albuminuria. Therefore, our patients were not adequately assessed for masked kidney dysfunction. Furthermore, we were unable to completely exclude the influence of drugs because we were unable to re-evaluate patients who had been taking angiotensin II receptor blockers, angiotensin-converting enzyme inhibitors, and MR antagonists before adrenalectomy since the diagnosis of PA without discontinuing these drugs again. However, we found that preoperative MR antagonist treatment was not a significant factor associated with a certain drop in eGFR, and there were no significant findings for angiotensin II receptor blockers and angiotensin-converting enzyme inhibitor medications. Discontinuing these drugs again in patients who are referred for adrenalectomy is not practical. This study included patients under these treatments to obtain preoperative real-world data.

In conclusion, 35.2% of the patients in this study experienced a certain drop in eGFR after adrenalectomy. A longer duration of hypertension, and lower preoperative serum potassium concentrations and serum potassium concentrations before potassium supplementation were identified as risk factors for a certain drop in eGFR after adrenalectomy in patients with PA. Our findings could be useful for surgeons to inform patients preoperatively about whether there is the possibility of downgrading their GFR category.

## Figures and Tables

**Table1:** Clinical characteristics of 54 patients with primary aldosteronism

Variable	All patients (*n*=54)	Certain drop in eGFR (*n*=19)	No certain drop in eGFR (*n*=35)	*p* value
Age (years)	52.2±10.3	55.1±11.0	50.7±9.9	0.148
Male sex, *n* (%)*	23 (42.5)	6 (31.5)	17 (48.5)	0.227
Body mass index (kg/m^2^)	24.7±3.6	23.4±3.5	25.4±3.6	0.056
Duration of hypertension (years)	10.6±8.2	14.0±7.7	8.8±8.1	**0.026**
Preoperative systolic BP (mmHg)	144.2±15.4	147.6±19.1	142.3±13.2	0.233
Preoperative diastolic BP (mmHg)	87.7±9.9	87.1±11.9	88.1±9.1	0.729
Smoker, *n* (%)*	17 (31.4)	4 (21.0)	13 (37.1)	0.224
Diabetes mellitus, *n* (%)*	11 (20.4)	3 (15.7)	8 (22.8)	0.538
Hyperlipidemia, *n* (%)*	9 (16.7)	4 (21.0)	5 (14.2)	0.523
Antihypertensive drugs except for an MR antagonist**, *n*	2 (0–5)	2 (1–5)	2 (0–5)	0.195
ARB/ACEi, *n* (%)*	18 (33.3)	6 (31.5)	12 (34.2)	0.840
Ca-blocker, *n* (%)*	51 (94.4)	19 (100)	32 (91.4)	0.189
α-blocker, *n* (%)*	14 (25.9)	6 (31.5)	8 (22.8)	0.484
β-blocker, *n* (%)*	8 (14.8)	4 (21.0)	4 (11.4)	0.341
Antihypertensive combination drug, *n* (%)*	6 (11.1)	2 (10.5)	4 (11.4)	0.919
Diuretics, *n* (%)*	2 (3.7)	0 (0)	2 (5.7)	0.288
MR antagonist, *n* (%)*	15 (27.8)	3 (15.7)	12 (34.2)	0.147
Potassium supplement, *n* (%)*	33 (61.1)	14 (73.6)	19 (54.2)	0.162
Preoperative PRA (ng/mL/h)	0.25±0.21	0.24±0.28	0.26±0.16	0.778
Preoperative PAC (pg/mL)	364.0±266.6	389.3±224.9	350.3±292.6	0.616
Preoperative serum sodium concentration (mmol/L)	142.5±2.1	143.3±1.8	142.2±2.2	0.069
Preoperative serum potassium concentration (mmol/L)	3.4±0.5	3.2±0.4	3.5±0.5	**0.009**
Serum potassium concentration before potassium supplementation (mmol/L)	2.8±0.5	2.4±0.4	2.9±0.5	**0.001**
Preoperative eGFR (mL/min/1.73 m^2^)	72.1±22.7	67.5±24.5	74.5±22.1	0.292
Preoperative serum creatinine concentration (mg/dL)	0.85±0.38	0.87±0.34	0.85±0.40	0.873
Preoperative serum total cholesterol concentration (mg/dL)	200.3±33.6	202.3±33.9	199.2±34.5	0.759

Data are shown as the mean±standard deviation, *number (percentage), or **median (range). BP, blood pressure; eGFR, estimated glomerular filtration rate; MR, mineralocorticoid receptor; ARB, angiotensin II receptor blocker; ACEi, angiotensin-converting enzyme inhibitor; PRA, plasma renin activity; PAC, plasma aldosterone concentration

**Table2:** Univariate and multivariate logistic regression analyses of factors associated with a certain drop in eGFR

Factors		Univariate analysis		Multivariate analysis	
		OR (95% CI)	*p* value	OR (95% CI)	*p* value
Age	years	1.044 (0.984–1.108)	0.151	0.984 (0.889–1.090)	0.761
Male sex	Male/female	0.489 (0.151–1.579)	0.231		
Body mass index	kg/m^2^	0.846 (0.709–1.009)	0.063	0.775 (0.590–1.017)	0.066
Duration of hypertension	years	1.082 (1.006–1.163)	**0.035**	1.173 (1.043–1.319)	**0.008**
Preoperative systolic BP	mmHg	1.023 (0.985–1.062)	0.234		
Preoperative diastolic BP	mmHg	0.990 (0.936–1.047)	0.723		
Smoker	Yes/no	0.451 (0.123–1.654)	0.230	0.711 (0.106–4.760)	0.725
Diabetes mellitus	Yes/no	1.726 (0.449–6.641)	0.427	6.726 (0.459–98.627)	0.164
Hyperlipidemia	Yes/no	1.600 (0.374–6.845)	0.526		
Antihypertensive drugs except for an MR antagonist	quantity	1.407 (0.838–2.362)	0.197		
MR antagonist	Yes/no	0.317 (0.077–1.301)	0.111		
Potassium supplement	Yes/no	2.358 (0.697–7.975)	0.168		
Preoperative PRA	ng/mL/h	0.625 (0.040–9.776)	0.737		
Preoperative PAC	pg/mL	1.001 (0.998–1.003)	0.611		
Preoperative serum sodium concentration	mmol/L	1.302 (0.973–1.743)	0.076		
Preoperative serum potassium concentration	mmol/L	0.203 (0.057–0.731)	**0.015**	0.145 (0.026–0.818)	**0.029**
Serum potassium concentration before potassium supplementation	mmol/L	0.124 (0.030–0.516)	**0.004**	0.111 (0.017–0.731)	**0.022**
Preoperative eGFR	mL/min/1.73 m^2^	0.986 (0.961–1.012)	0.289	0.991 (0.951–1.034)	0.684
Preoperative serum creatinine concentration	mg/dL	1.128 (0.264–4.815)	0.870		
Preoperative serum total cholesterol concentration	mg/dL	1.003 (0.986–1.020)	0.754		

CI, confidence interval; OR, odds ratio; BP, blood pressure; MR, mineralocorticoid receptor; PRA, plasma renin activity; PAC, plasma aldosterone concentration; eGFR, estimated glomerular filtration rate

## Data Availability

Restrictions apply to the availability of some or all data generated or analyzed during this study to preserve patient confidentiality or because they were used under license. The corresponding author will on request detail the restrictions and any conditions under which access to some data may be provided.
