# Supplementary material for: Changes in kidney function after adrenalectomy in patients with primary aldosteronism
Source: Fujita Med J. 2024 Oct 31;11(1):28–35. doi: 10.20407/fmj.2024-011 (PMC11782942; doi:10.20407/fmj.2024-011)
Supplement: Supplementary file 1 — Supplementary Figure [file fmj-11-028-s001.pdf]

Supplementary Figure

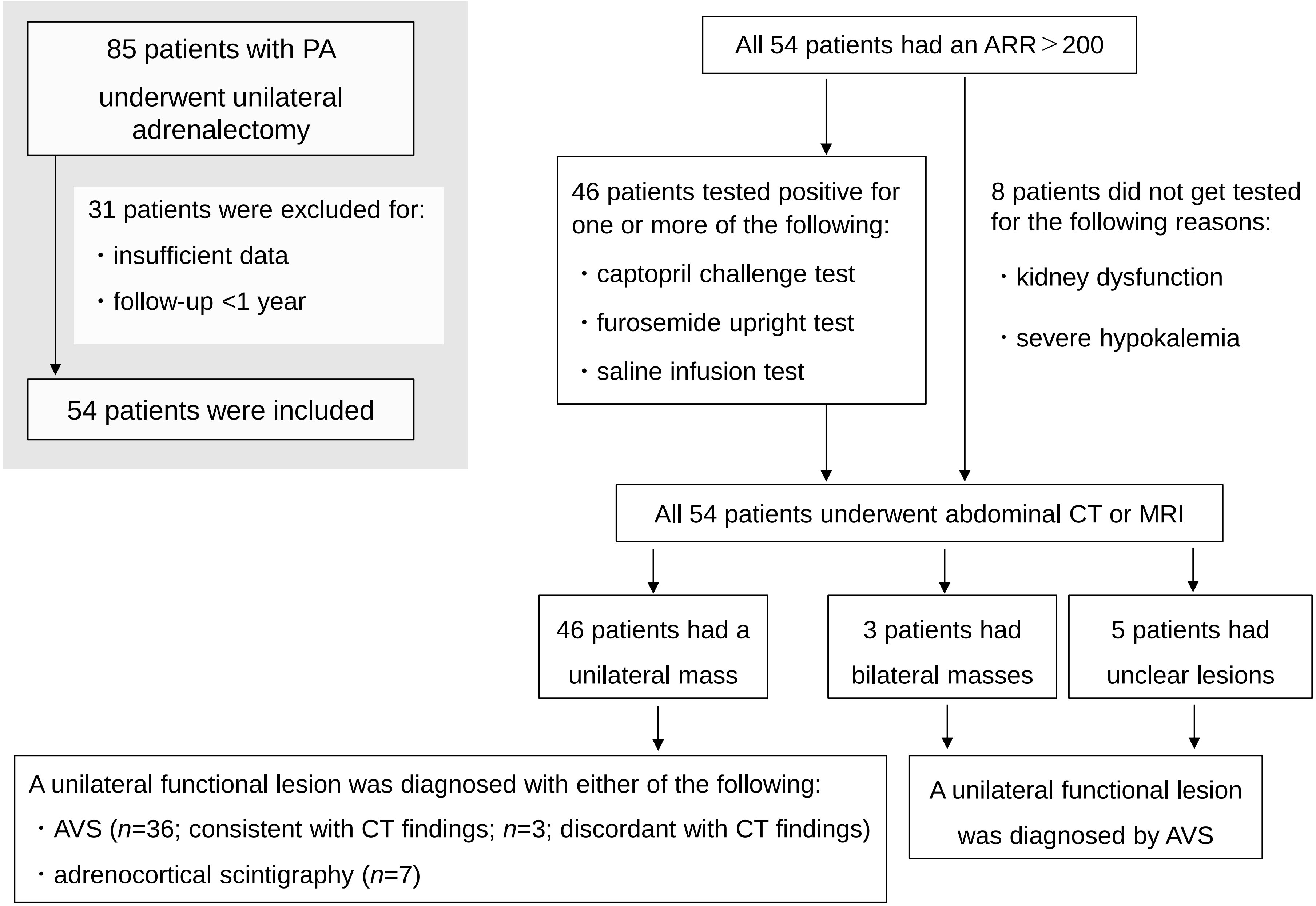

Supplementary Figure

Flowchart of the patients' selection and the diagnostic process. PA, primary aldosteronism; ARR, aldosterone-to-renin ratio; CT, computed tomography; MRI, magnetic resonance imaging; AVS, adrenal venous sampling
